# Moxibustion ameliorates chronic inflammatory visceral pain via spinal circRNA-miRNA-mRNA networks: a central mechanism study

**DOI:** 10.1186/s13041-024-01093-7

**Published:** 2024-05-15

**Authors:** Dan Zhang, Xiaoqing Dong, Xiaoying Li, Yanting Yang, Hongna Li, Yue Hong, Guang Yang, Xiehe Kong, Xuejun Wang, Xiaopeng Ma

**Affiliations:** 1 Laboratory of Acupuncture-Moxibustion and Immunology, Shanghai Research Institute of Acupuncture and Meridian, Shanghai University of Traditional Chinese Medicine, Shanghai, 200030 China; 2 Yueyang Hospital of Integrated Traditional Chinese and Western Medicine, Shanghai University of Traditional Chinese Medicine, Shanghai, 200437 China; 3 Department of Acupuncture and Moxibustion, Xi’an Hospital of Encephalopathy, Shaanxi University of Chinese Medicine, Shaanxi, 710032 China; 4 Department of Acupuncture and Moxibustion, Shanghai Municipal Hospital of Traditional Chinese Medicine, Shanghai University of Traditional Chinese Medicine, Shanghai, 200071 China; 5 Eye Institute and Department of Ophthalmology, Eye & ENT Hospital, Fudan University, Shanghai, 200031 China

**Keywords:** Visceral pain, Colitis, Moxibustion, Analgesia, Central mechanism, ceRNA network

## Abstract

This study aimed to unveil the central mechanism of moxibustion treating chronic inflammatory visceral pain (CIVP) from the angle of circRNA-miRNA-mRNA networks in the spinal cord. The rat CIVP model was established using a mixture of 5% (w/v) 2,4,6-trinitrobenzene sulfonic acid and 50% ethanol at a volume ratio of 2:1 via enema. Rats in the moxibustion group received herb-partitioned moxibustion at Tianshu (ST25, bilateral) and Qihai (CV6) points. The abdominal withdrawal reflex (AWR), mechanical withdrawal threshold (MWT), and thermal withdrawal latency (TWL) were adopted for pain behavior observation and pain sensitivity assessment. The circRNA, miRNA, and mRNA expression profiles were detected using the high-throughput sequencing technique. Relevant databases and bioinformatics analysis methods were used to screen for differentially expressed (DE) RNAs and build a circRNA-miRNA-mRNA (competing endogenous RNA) ceRNA regulatory network. The real-time quantitative PCR was employed to verify the sequencing result. CIVP rat models had a significantly higher AWR and lower TWL and MWT than normal rats. Between normal and model rats, there were 103 DE-circRNAs, 16 DE-miRNAs, and 397 DE-mRNAs in the spinal cord. Compared with the model group, the moxibustion group had a lower AWR and higher TWL and MWT; between these two groups, there were 118 DE-circRNAs, 15 DE-miRNAs, and 804 DE-mRNAs in the spinal cord. Two ceRNA networks were chosen to be verified. As a result, moxibustion’s analgesic effect on visceral pain in CIVP rats may be associated with regulating the circRNA_02767/rno-miR-483-3p/Gfap network in the spinal cord and improving central sensitization.

**Supplementary Information:**

The online version contains supplementary material available at 10.1186/s13041-024-01093-7.

## Introduction

Visceral pain (VP) is the pain deriving from internal organs and is also one of the most common types of chronic pain experienced by inflammatory bowel disease (IBD) patients [1]. A hospital cohort study showed that 38% of IBD patients suffered from chronic pain, and 91% suffered abdominal pain; they usually had a high disease activity, hindered quality of life (QOL), and negative moods like anxiety and depression [2]. Abdominal pain caused by chronic and recurrent gastrointestinal inflammation, a chronic inflammatory visceral pain (CIVP), is a major type of IBD-associated VP [3, 4]. Hence, relieving pain and improving QOL are important demands of IBD patients. To date, short-term and small-dose painkillers such as non-steroidal anti-inflammatory drugs, antidepressants, cyclooxygenase-2 inhibitors, and psychoactive agents such as marijuana and opioids are often recommended to manage abdominal pain in IBD patients [5, 6]. However, aside from side effects and addiction, long-term use of opioids has been proven to be associated with poor QOL and a high mortality rate [7–9]. Therefore, clinical management of IBD-related CIVP is in high demand; seeking safe, effective analgesic approaches or drugs has become the focus of clinical staff and patients. It’s been verified that moxibustion, a non-pharmaceutical therapy, can reduce abdominal pain in Crohn’s disease (CD), ulcerative colitis (UC), and irritable bowel syndrome (IBS) and improve QOL and anxiety [10–15]. However, moxibustion’s analgesic mechanism is still unclear.

The sensitization of peripheral and central nervous systems is an essential mechanism in developing IBD-associated CIVP [16–18]. Research shows that CIVP patients and animal models all present persistent visceral hyperalgesia, which involves multiple abnormal epigenetic modifications in the spinal cord and brain [19–21]. Circular RNAs (circRNAs) are closed-loop RNA molecules with microRNA (miRNA, miR) response element (MRE) structures. They are highly conservative, extensively distributed, and tissue-specific and act as the key competing endogenous RNA (ceRNA) of epigenetic regulatory factors [22, 23]. Recent studies have found that circRNAs play an important role in chronic pains, including lumbago and neuropathic pain in degenerative diseases [24–27]. As the “molecular sponge” of miRNAs, circRNAs can competitively bind with miRNAs to regulate the mRNA transcription of target genes; these three groups of RNAs interact and build a ceRNA network [28]. Some miRNAs and the corresponding target genes participate in the regulation of pain-related gene expression in the central nervous system [29, 30]. It is unknown and worth discovering whether moxibustion improves visceral hyperalgesia and treats CIVP by modulating the ceRNA network in the spinal cord. So, in this study, we used 2,4,6-trinitrobenzene sulfonic acid (TNBS) to develop a rat CIVP model. With this model, we screened for differentially expressed (DE) circRNAs, miRNAs, and mRNAs in the spinal cord using the full-transcriptome high-throughput sequencing technique and built circRNA-miRNA-mRNA ceRNA networks via databases such as miRanda and bioinformatics analysis techniques. This study was supposed to reveal the central mechanism of moxibustion treating IBD-associated CIVP from the perspective of spinal cord ceRNA network regulation and to provide novel ideas for moxibustion’s analgesic mechanism research.

## Results

### Comparison of AWR(Abdominal withdrawal reflex), MWT(Mechanical withdrawal threshold), and TWL(Thermal withdrawal latency)

**Fig. 1:**
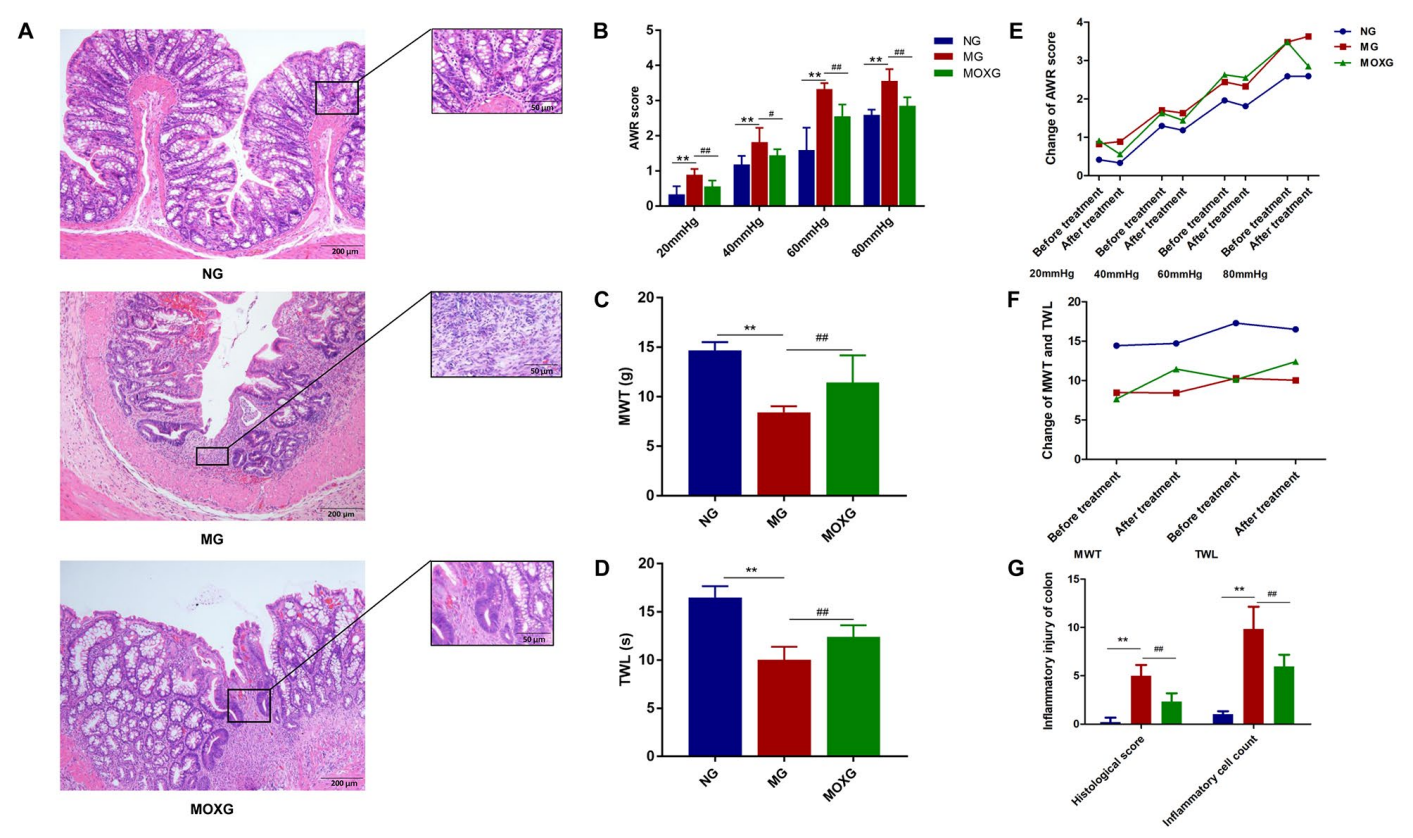
**Histomorphological observation of rat colonic tissue and pain behaviors** (**A**) Hematoxylin-eosin (HE) staining of colonic tissue (×100, ×400);(**B**) Comparison of the AWR at rectal distension pressures of 20, 40, 60, and 80 mmHg; (**C**) Comparison of the MWT; (**D**) Comparison of the TWL; (**E**) Change of the AWR pre and post treatment at rectal distension pressures of 20, 40, 60, and 80 mmHg; (**F**) Change of the MWT and TWL pre and post treatment; (**G**) Comparison of the histological score and inflammatory cell count. vs. NG, ^**^P<0.01; vs. MG, ^##^*P*<0.01. NG: Normal group; MG: Model group; MOXG: Moxibustion group. AWR: Abdominal withdrawal reflex; MWT: Mechanical withdrawal threshold; TWL: Thermal withdrawal latency

Rats in the model group (MG) had a higher AWR and lower MWT and TWL at each level of rectal distension pressure compared with the normal group (NG) (all *P*<0.01). In the MG, rats showed severe colonic damage, manifesting as loss of colonic mucosal epithelium, ulcers reaching the submucosal and muscular layers, irregular gland arrangement or even loss of glands, and edema of submucosal connective tissue with extensive inflammatory cell infiltration. Compared with the MG, the AWR dropped (*P*_40mmHg_<0.05, *P*_other pressure levels_<0.01), and the MWT and TWL increased (all *P*<0.01) at each pressure level in the moxibustion group (MOXG). Moreover, in the MOXG, healed ulcers were found, together with slightly irregularly arranged swelling glands and mild submucosal edema and inflammatory cell infiltration (Fig. 1).

### Changes in the profiling of circRNA, miRNA, and mRNA in rat spinal cord

**Fig. 2:**
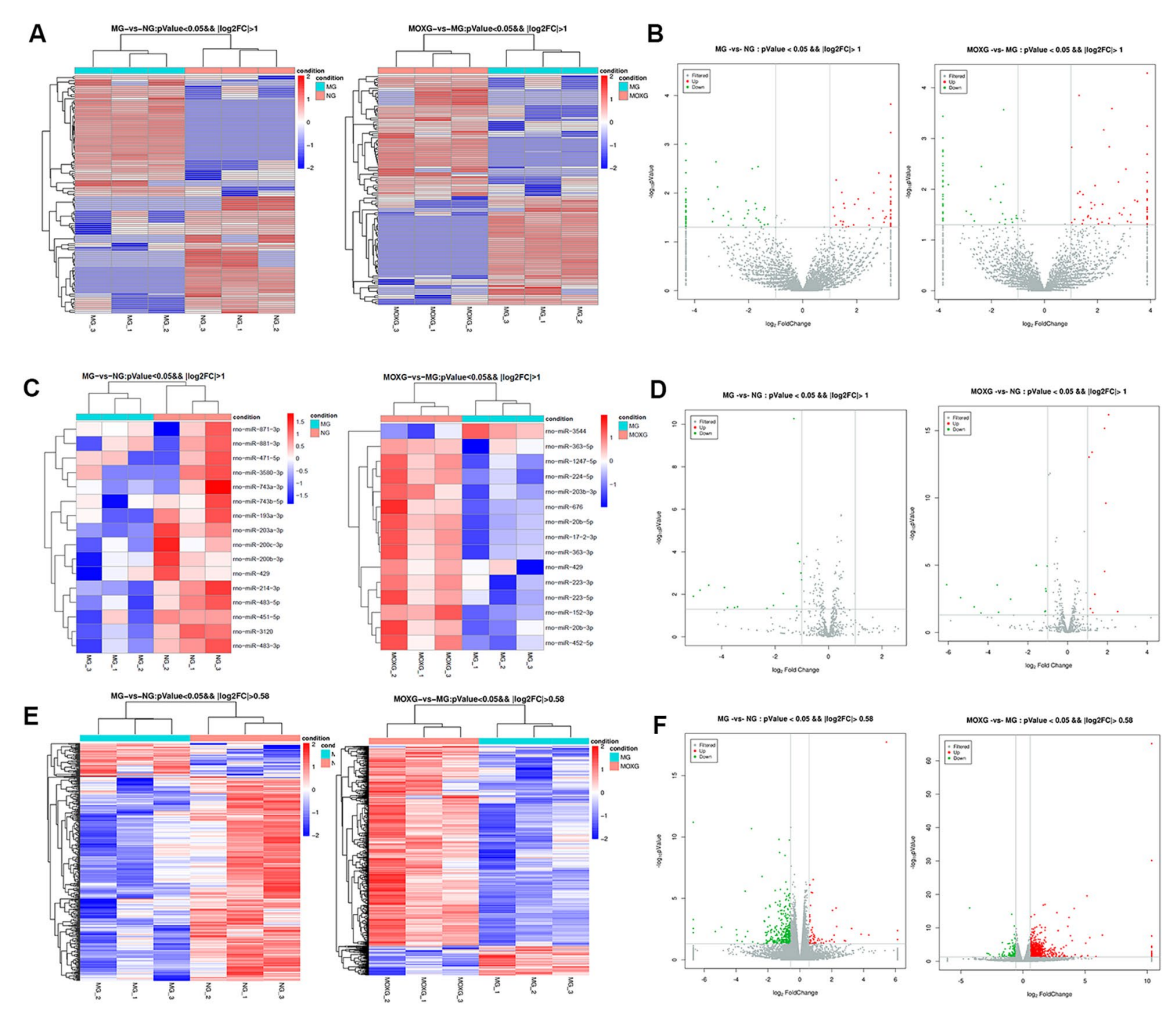
**Heat map and volcano map of DE circRNAs, miRNAs, and mRNAs** (**A**) Heat map of DE circRNAs (MG vs. NG); heat map of DE circRNAs (MOXG vs. MG). (**B**) Volcano map of DE circRNAs (MG vs. NG); heat map of DE circRNAs (MOXG vs. MG). (**C**) Heat map of DE miRNAs (MG vs. NG); heat map of DE miRNAs (MOXG vs. MG). (**D**) Volcano map of DE miRNAs (MG vs. NG); volcano map of DE miRNAs (MOXG vs. MG). (**E**) Heat map of DE mRNAs (MG vs. NG); heat map of DE mRNAs (MOXG vs. MG). (**F**) Volcano map of DE mRNAs (MG vs. NG); volcano map of DE mRNAs (MOXG vs. MG). *n* = 3. On heat maps, red represents up-regulation, and blue means down-regulation; the darker the color, the more significant the change. On volcano maps, red represents significantly up-regulated RNAs, green indicates significantly down-regulated RNAs, and grey means insignificantly differentially expressed RNAs. NG: Normal group; MG: Model group; MOXG: Moxibustion group. DE: Differentially expressed

Compared with the NG, 103 DE-circRNAs (56 up-regulated/47 down-regulated), 16 DE-miRNAs (all down-regulated), and 397 DE-mRNAs (58 up-regulated/339 down-regulated) were found in the spinal cord tissue of MG rats. Compared with the MG, 118 DE-circRNAs (59 up-regulated/59 down-regulated), 15 DE-miRNAs (14 up-regulated/1 down-regulated), and 804 DE-mRNAs (703 up-regulated/101 down-regulated) were found in the spinal cord tissue of MOXG rats. Cluster analysis was performed for the DE-circRNAs, miRNAs, and mRNAs (Fig. 2).

### Constructing the circRNA-miRNA-mRNA ceRNA networks in the spinal cord and screening

**Fig. 3:**
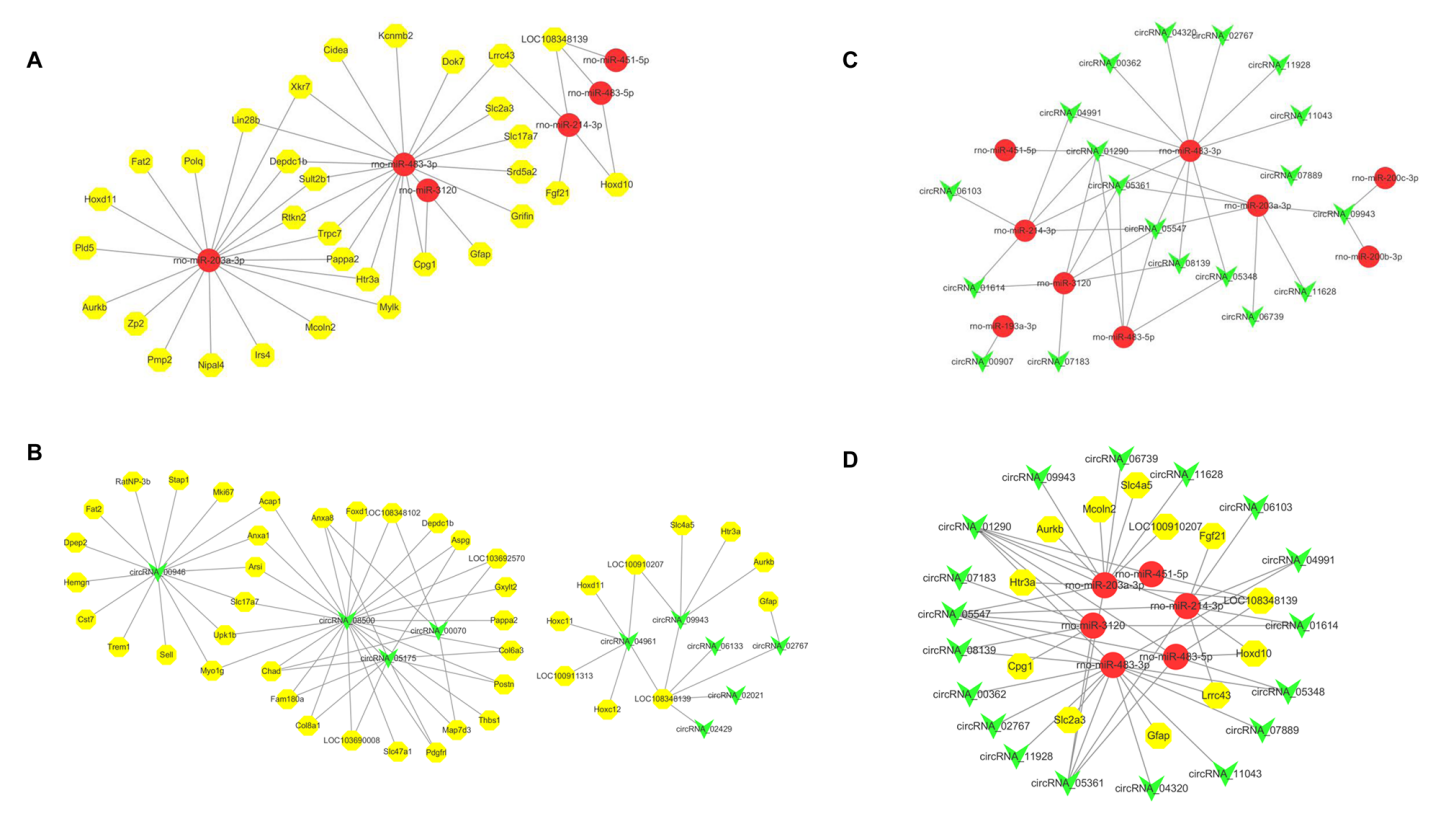
**Interacting circRNA-miRNA, miRNA-mRNA, circRNA-mRNA pairs and circRNA-miRNA-mRNA networks** (**A**): CircRNA-miRNA pairs, in which circRNAs were all down-regulated, and miRNAs were up-regulated. (**B**): MiRNA-mRNA pairs, in which miRNAs were all up-regulated, and mRNAs were down-regulated. (**C**): CircRNA-mRNA pairs, in which circRNAs and mRNAs were all down-regulated. (**D**): CircRNA-miRNA-mRNA networks. Green represents circRNAs, red represents miRNAs, and yellow indicates mRNAs. DE: differentially expressed

Based on the Pearson correlation analysis, miRanda v3.3a was used to predict the target pairs among 3 DE-RNAs. Finally, we discovered 38 cicRNA-miRNA negative regulation pairs, 46 miRNA-mRNA negative regulation pairs, and 2762 circRNA-miRNA positive regulation pairs (MG vs. NG). We then used CytoScape 3.6.1 to sketch the circRNA-miRNA-mRNA ceRNA networks, including 19 circRNAs, 6 miRNAs, and 12 mRNAs (Supplementary Tables 1, 2, 3, 4; Fig. 3). And, the top 6 ceRNA networks were circRNA_04991/rno-miR-214-3p/Lrrc4, circRNA_04991/rno-miR-483-3p/Lrrc4, circRNA_01290/rno-miR-214-3p/LOC108348139, circRNA_01290/rno-miR-451-5p/LOC108348139, circRNA_01290/rno-miR-203a-3p/LOC108348139, and circRNA_01290/rno-miR-483-5p/LOC108348139.

### Target gene functional annotation and enrichment analysis

**Fig. 4:**
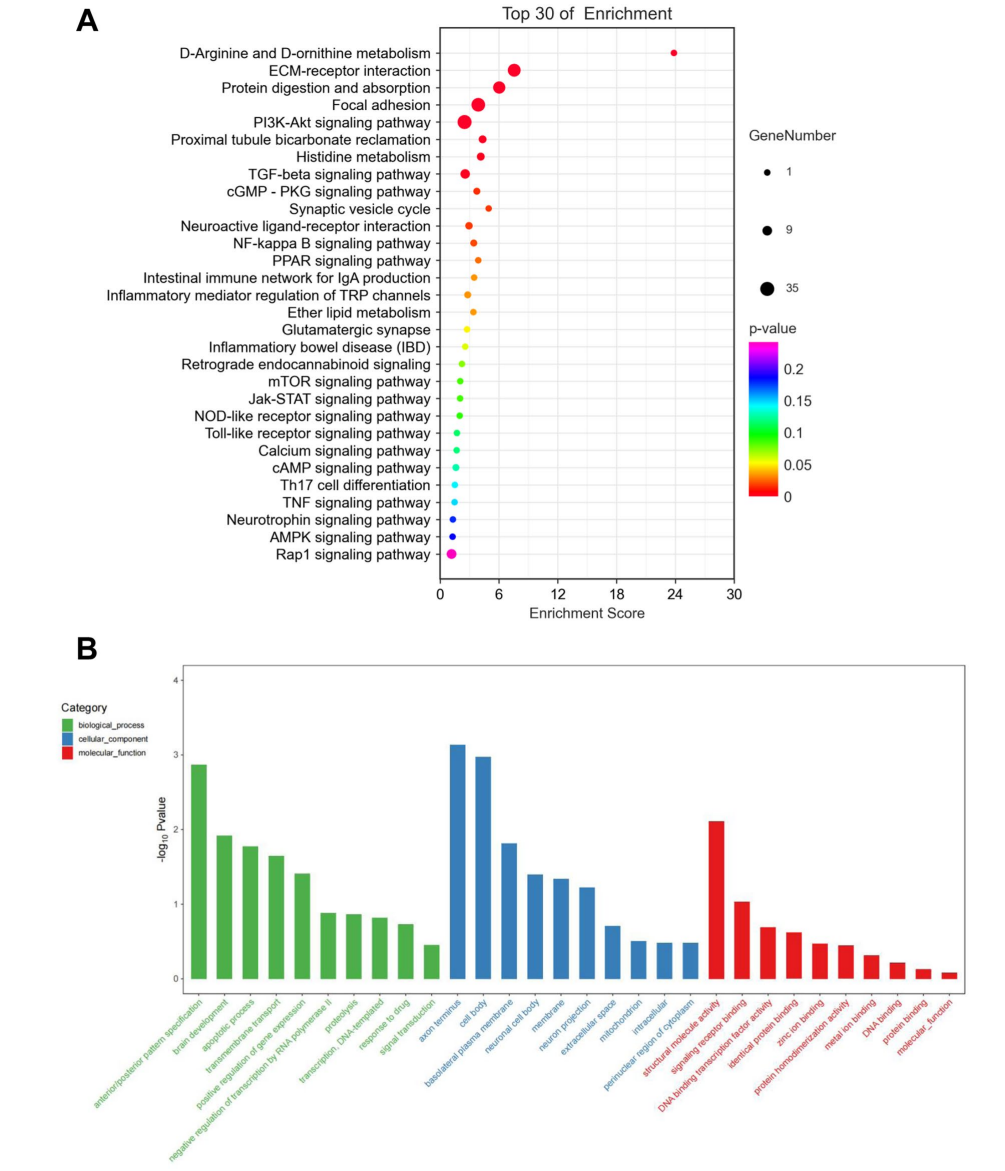
Target gene function and pathway enrichment analyses (**A**): KEGG pathway enrichment. The size of the dots corresponds to the number of differentially expressed genes in GO items. The enrichment *P*-value shrinks, and the significance grows as the dot’s color changes from purple to blue, green, and red. (**B**): GO function enrichment. Green represents biological processes, blue indicates cellular components, and red means molecular functions

Functional annotation and enrichment analysis of the corresponding DE-mRNAs were conducted using KEGG and GO databases (Supplementary Table 5). It’s revealed that the top 5 enriched signaling pathways were D-Arginine and D-ornithine metabolism, ECM-receptor interaction, protein digestion and absorption, focal adhesion, and PI3K-Akt pathways. Of the top 30 enriched signaling pathways, some were related to pain, such as cGMP-PKG, NF-κB, and mTOR signaling pathways, and some were associated with inflammation, such as Jak-STAT, NOD-like receptor, and TOLL-like receptor, and cAMP signaling pathways (Fig. 4A). The involved biological processes mainly included anterior/posterior pattern specification, brain development, apoptotic process, transmembrane transport, positive regulation of gene expression, negative regulation of transcription by RNA polymerase II, and protein hydrolysis. The cell components included axonal terminals, cell bodies, basolateral plasma membranes, and neuronal cell bodies. Molecular functions included structural molecular activity, signaling receptor binding, DNA binding transcription factor activity, protein recognition binding, zinc ion binding, etc. (Fig. 4B).

### Verification of sequencing results

**Fig. 5:**
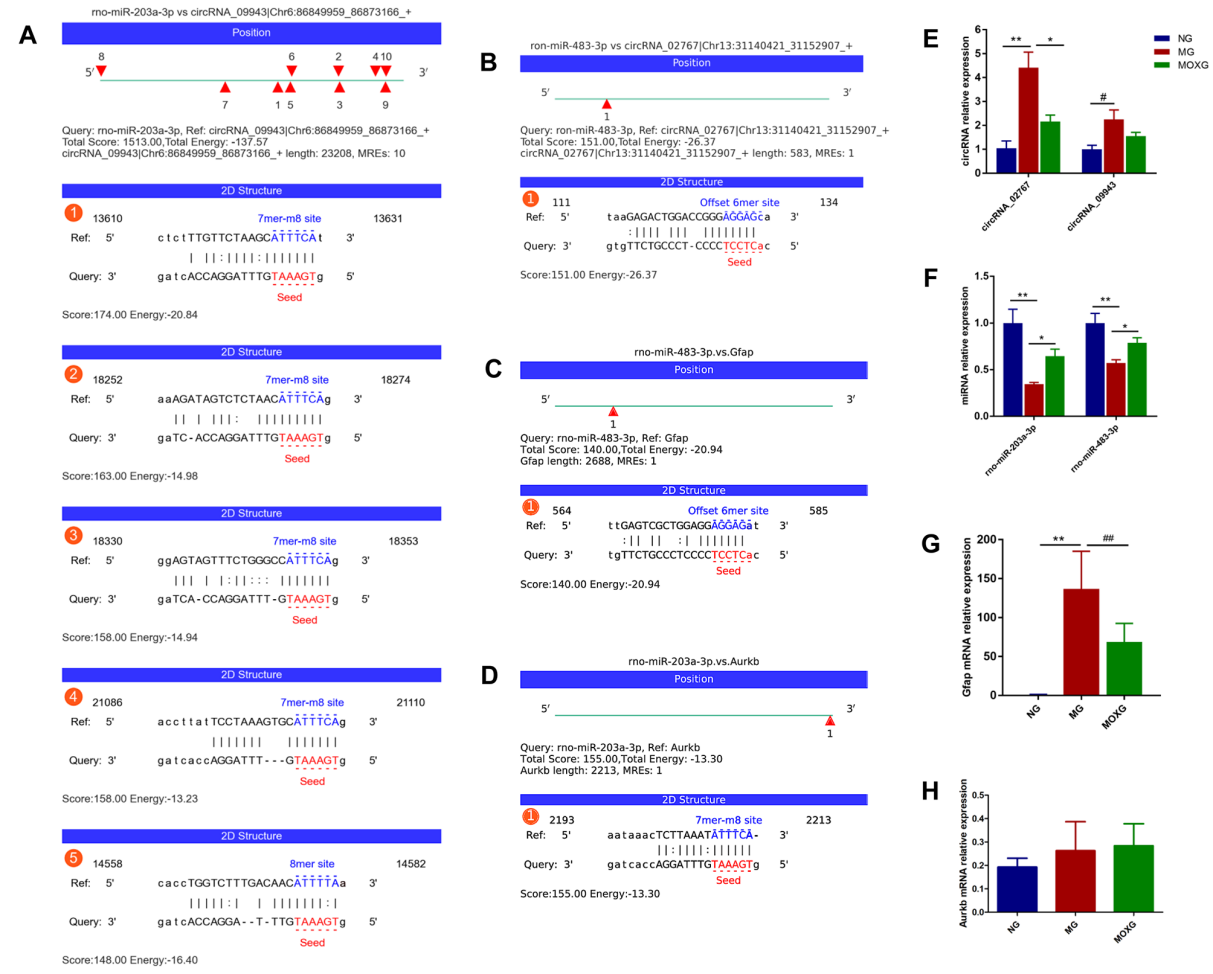
**Prediction of ceRNA network binding sites and qRT-PCR verification** (**A**): Predicted binding sites of circRNA_09943 and rno-miR-203a-3p; (**B**): Predicted binding sites of circRNA_02767 and rno-miR-483-3p; (**C**): Predicted binding sites of rno-miR-203a-3p and Aurkb mRNA; (**D**): Predicted binding sites of rno-miR-483-3p and Gfap mRNA. Red triangles represent the binding regions of miRNAs (MRE). (**E**): Comparison of the relative expression of circRNA_0994 and circRNA_02767 (**F**): Comparison of the relative expression of rno-miR-203a-3p and rno-miR-483-3p; (**G**): Comparison of the relative expression of Gfap mRNA; (**H**): Comparison of the relative expression of Aurkb mRNA. *n* = 6. vs. NG, ^**^P<0.01; vs. MG, ^*^P<0.05, ^##^*P*<0.01. NG: Normal group; MG: Model group; MOXG: Moxibustion group

Two DE pairs, circRNA_09943/rno-miR-203a-3p and circRNA_02767/rno-miR-483-3p, were selected by analyzing the intersection between two groups of circRNA-miRNA pairs (MG vs. NG and MOXG vs. MG). Then, the mRNAs co-expressed in the three groups were screened for the target genes of the above two miRNAs, given the premise of pain-related, and we got two ceRNA networks: circRNA_02767/rno-miR-483-3p/Gfap and circRNA_09943/rno-miR-203a-3p/Aurkb, which were then verified, and possible binding sites were predicted (Fig. 5A, B, C, D). The results presented that, compared with the NG, rats in the MG showed a significant increase in the expression of circRNA_02767 and circRNA_09943, a notable decrease in the expression of miR-203a-3p and miR-483-3p, and elevated Gfap mRNA expression in the spinal cord tissue (all *P*<0.01), while the Aurkb mRNA content only had an increasing tendency without statistical significance (*P*>0.05). Compared with the MG, in the spinal cord tissue of MOXG rats, the expression of circRNA_02767 was down-regulated, the expression of miR-203a-3p and miR-483-3p was up-regulated, and the expression of Gfap mRNA was reduced (all *P*<0.05), while the expression of circRNA_09943 and Aurkb mRNA showed a statistically insignificant decreasing tendency (both *P*>0.05) (Fig. 5E, F, G, H).

## Discussion

Abdominal pain is the primary main recurrent clinical manifestation during the disease course in IBD patients [31]. When inflammation attacks, intestinal efferent nerve endings activated by inflammation mediators often cause persistent pain [32]. Nevertheless, even when the intestinal inflammation is in remission, 30-50% of the population still suffer from severe abdominal pain, and in some cases, the pain “transfers” from the intestine to skin or other visceral regions, which hampers the QOL and increases the risk of pressure, anxiety, and depression [33, 34]. Thus, safe and effective management of pain and improving QOL have become highly demanded among IBD patients. Aside from drugs for ameliorating intestinal lesions, analgesics like opioids are commonly used [35]. However, long-term use or large doses of opioid drugs has been found related to the increase in mortality and infection in IBD patients, thus triggering opioid use disorders [36, 37]. Therefore, some scholars point out that it is indeed crucial to find a replacement drug or therapy for IBD patients suffering from pain [38]. As a non-pharmaceutical external therapy, acupuncture-moxibustion is simple, mild, and multi-targeting [39, 40]. In recent years, numerous studies have verified the positive effect of moxibustion on abdominal pain. For example, moxibustion can mitigate abdominal pain in CD by reducing its intensity, frequency, and duration; moxibustion can also improve abdominal pain, bloating, and mental symptoms by regulating the brain-gut axis function in constipation-dominant IBS patients [41, 42]. In this study, moxibustion reduced the AWR and increased the MWT and TWL, lowering hyperalgesia, in TNBS-induced CIVP rat models, suggesting the analgesic effect of moxibustion on CIVP rats, which is in line with previous reports [43, 44]. It inspires that moxibustion may become an analgesic replacement and be applied in the routine treatment of IBD patients suffering from pain. Further research on moxibustion’s analgesic mechanism can provide reliable scientific evidence for the clinical application.

CircRNAs are differentially expressed in various regions or subcellular neuronal structures. They play a role in modulating brain development, neuronal differentiation, and synaptic plasticity and act as important regulatory factors in the occurrence and development of neurological diseases such as Parkinson’s disease and Alzheimer’s disease [45–47]. Some studies hold that circRNAs may be involved in regulating chronic pain, including the development of pain and central sensitization [25, 48]. Chen et al [49] proved that spinal cord circKcnk9 mediated IBS-associated chronic visceral hyperalgesia. However, research is still limited on circRNAs and IBD-related CIVP. Based on the above findings, the current study adopted an IBD-related CIVP model to investigate the action mechanism of circRNAs in CIVP. The results suggested that compared with the NG, there were 103 DE circRNAs (59 up-regulated; 47 down-regulated) in the spinal cord of MG rats; compared with the MG, 118 DE circRNAs (59 up-regulated; 59 down-regulated) were found in the spinal cord of MOXG rats. Some DE circRNAs between the MOXG and MG participate in pain conduction, e.g., sodium ion channel proteins Slc8a1 and Slc8a3, inflammation-related signaling molecule Lilrb3l, Ubr3, Stx8, Zfp423, Myt1l, etc. (Supplementary Table 6). The results indicated that abnormally expressed circRNAs in the spinal cord were involved in the development of CIVP; moxibustion might relieve hyperalgesia in CIVP rats by regulating circRNAs in the spinal cord.

CircRNAs competitively bind with miRNAs through MRE to release miRNAs’ suppression on target genes and regulate the transcription of target mRNAs [50]. In this study, we discovered 10 DE miRNAs co-expressed in the three groups: rno-miR-429, rno-miR-223-5P, rno-miR-203a-3p, rno-miR-450b-3p, rno-miR-214-3p, rno-miR-3120, rno-miR-322-3p, rno-miR-199a-5p, rno-miR-345-5p, and rno-miR-542-5p. It’s found that miRNAs play a role in visceral pain by modulating target gene transcription of peripheral or central neurotransmitters and their related proteins [51]. Our findings partly correlate with the previous research. For example, rno-miR-223-5P participates in modulating the polarization of microglia and plays an essential role in pain responses; the expression of spinal cord miR-214-3p can suppress astrocytes’ reactions and attenuate neuroinflammation and pain behavior in spinal nerve ligation (SNL) model rats [52–54]. In the current study, these miRNAs were down-regulated in the MG but up-regulated in the MOXG. Furthermore, we performed KEGG analysis for the target genes based on the negative regulation relationship between miRNAs and mRNAs. The analysis discovered pain-related signaling pathways such as PI3K-Akt, cGMP-PKG, NF-кB, and mTOR pathways and inflammation-related signaling pathways such as Jak-STAT, NOD-like receptor, TOLL-like receptor, and cAMP pathways, which were all highly enriched. The GO analysis revealed that the molecular functions of the target gene were mainly enriched in structural molecule activity, signal receptor binding, DNA-binding transcription factor activity, protein recognition binding, zinc ion binding, etc. These findings suggest that moxibustion may ease pain by regulating miRNAs’ activity via circRNAs to modulate the target mRNA transcription and pertinent signaling pathways.

As the term “ceRNA” arises, circRNAs and other noncoding RNAs are endowed with novel biological functions, and the potential regulation mechanisms of transcription are also extended [55]. In a study, the lncRNA/circRNA-miRNA-mRNA ceRNA networks were discovered in neurological diseases mediated by microglia and astrocytes [56]. In the current study, we observed the whole transcriptome expression profiling of CIVP rats’ spinal cords using the high-throughput sequencing technique and constructed circRNA-miRNA-mRNA ceRNA networks to discuss the mechanism of moxibustion treating IBD-related CIVP. We verified two networks out of numerous ceRNA networks, circRNA_09943/rno-miR-203a-3p/Aurkb and circRNA_02767/rno-miR-483-3p/Gfap. In these two networks, miR-203a-3p and miR-483-3p may relate to pain, and two target genes, Aurkb and Gafp are also found participating in pain regulation [57, 58]. Aurkb is a serine/threonine protein kinase that modulates the separation of chromosomes and cytoplasm during mitosis and is a key protein in the cell cycle signaling pathway. Shen et al [59] found that Aurkb was essential in spinal microglial proliferation and neuropathic pain, and modulating Aurkb might be an effective approach in treating peripheral nerve injury-related neuropathic pain. Gfap is an important immune marker of astrocytes, which can encourage the occurrence of IBS-related abdominal pain [60, 61]. Pro-inflammatory cytokines and chemokines produced by astrocytes and microglia are crucial in inducing and maintaining central sensitization [62]. According to PCR verification, compared with the MG, circRNA_02767 was down-regulated, rno-miR-203a-3p and rno-miR-483-3p were up-regulated, and Gfap mRNA was down-regulated in the spinal cord of MOXG rats (all *P*<0.05), while circRNA_09943 and Aurkb mRNA only showed a decreasing tendency (*P*>0.05). The circRNA-miRNA-mRNA network plays a crucial role in IBD related CIVP and moxibustion may improve pain hypersensitivity in rats and alleviate CIVP by regulating the spinal cord circRNA 02767/rno miR-483-3p/Gfap network.

## Conclusion

This study analyzed, predicted, and constructed ceRNA networks by the high-throughput sequencing technique and used miRanda software to select two ceRNA networks to verify, circRNA_09943/rno-miR-203a-3p/Aurkb and circRNA_02767/rno-miR-483-3p/Gfap, which were closely associated with IBD-related CIVP. This study unveiled the central sensitization in CIVP and possible mechanisms of moxibustion analgesia. We proved that moxibustion could improve hyperalgesia in CIVP rats, and this effect might be achieved by regulating the spinal circRNA_02767/rno-miR-483-3p/Gfap ceRNA network.

However, some limitations should be mentioned here. This experiment only verified for pain-related ceRNA networks chosen based on the miRNAs co-expressed in the three groups. Therefore, further validation and exploration are needed for ceRNA networks with higher scores in future research. Meanwhile, due to the large amount of full transcription sequencing data and the differences in screening methods, other interesting research results remain to be discovered. Secondly, further analysis and confirmation of circRNA-miRNA and miRNA-mRNA interactions are required using experimental techniques such as dual luciferase reporter assay and pull down experiment. In addition, this experiment used the entire spinal cord sample for sequencing. So, exactly which cells in the spinal cord execute the regulation of the ceRNA network is also a question worthy of attention and research.

## Method and material

### Experimental animal

**Fig. 6:**
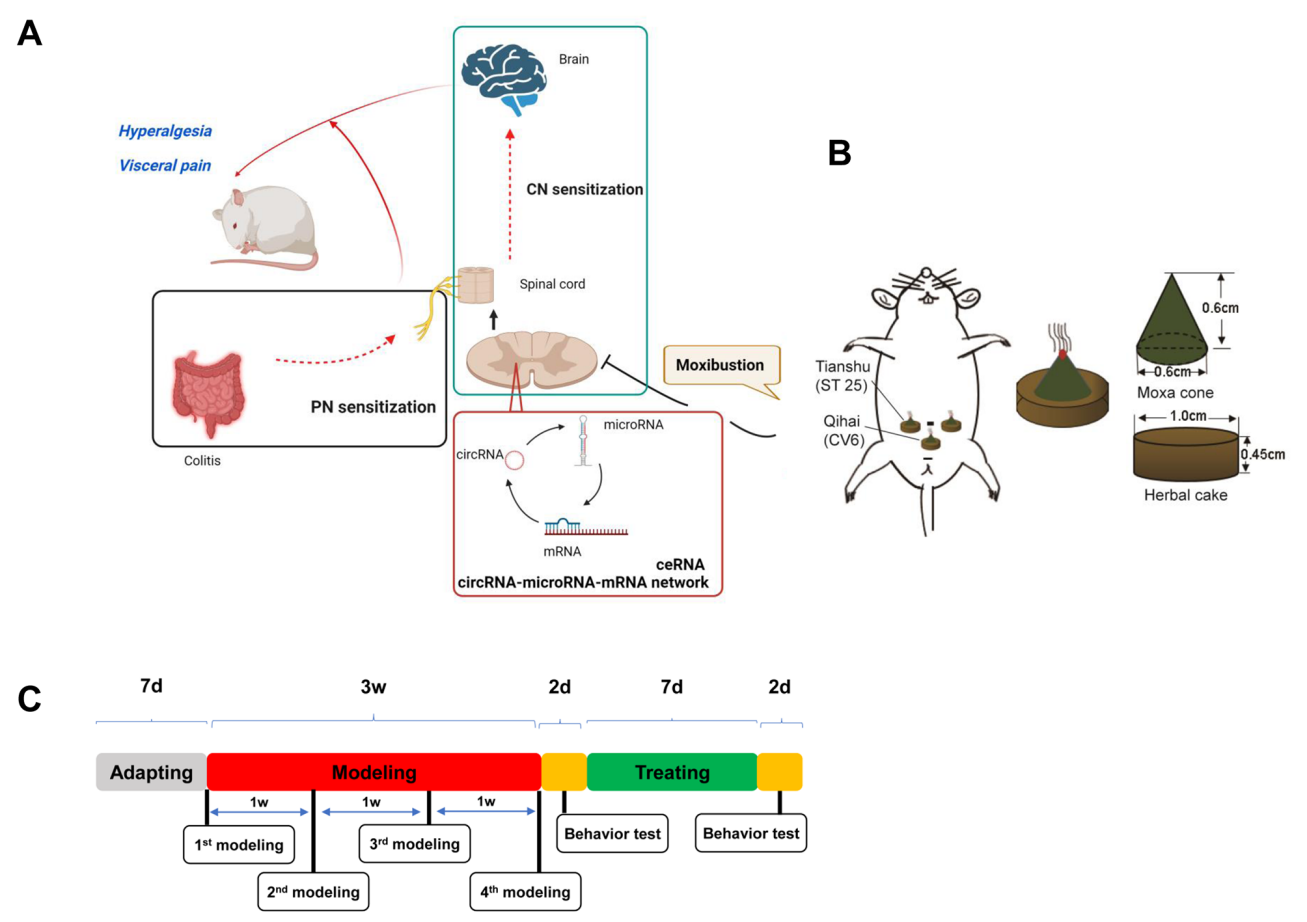
Illustration (**A**): Mechanism diagram; (**B**): Moxibustion (harb-partitioned moxibustion); (**C**): Experiment schedule and time points. CN: Central nerve; PN: Peripheral nerve; AWR: Abdominal withdrawal reflex; MWT: Mechanical withdrawal threshold; TWL: Thermal withdrawal latency

A total of 27 healthy SPF (Specific pathogen free) male Sprague-Dawley (SD) rats weighing (150 ± 20) g were supplied by Shanghai Jihui Experimental Animal Co., Ltd. (SCXK(Hu)2017-0012). Before the experiment started, rats were fed in an environment with the room temperature at (20 ± 2) °C and humidity at 50-70% for 7 d. This experiment was approved by the Ethics Committee of Yueyang Hospital of Integrated Traditional Chinese and Western Medicine (No.YYLAC-2020-085), and all procedures were in line with the Guiding Opinions on the Treatment of Experimental Animals issued by the Ministry of Science and Technology of the People’s Republic of China ((2006)398). Rats were randomized into a normal group , a model group, and a moxibustion group, 9 rats each. The experimental design and whole schedule are respectively shown in Fig. 6A and C.

### Establishment of CIVP model

The IBD-associated CIVP rat model was established via enema with the mixture of 5% (w/v) TNBS (Sigma, MO, USA) and 50% ethanol at a ratio of 2:1 [63, 64]. When the modeling procedure ended, each group of rats underwent pain behavior tests, and each group randomly donated one rat for pathological observation of colonic tissues to verify the modeling result.

### Intervention

Rats in the MOXG received herb-partitioned moxibustion (HPM) at Tianshu (ST25, bilateral) and Qihai(CV6) points (Fig. 6B), 2 cones for each point each session, once daily for 7 d. Herbal cakes (1 cm in diameter and 0.45 cm high) used in the HPM intervention were mainly made with aconite powder (Huaji Pharmaceutical Industry, Shanghai, China) mixed with yellow rice wine. Each moxa cone (0.6 cm in diameter and 0.6 cm high) used in the intervention was made of 90 mg fine moxa wool (Nanyang Hanyi Moxa Wool Co., Ltd., Henan, China). Rats in the NG and MG did not receive the intervention.

### Pain behavior tests and pain sensitivity assessment

#### AWR score

Visceral sensitivity was measured by referring to AI-Chaer’s [65] method using rectal dilation stimulation with the pressure going from 20 mmHg, 40 mmHg, 60 mmHg, to 80 mmHg. Each rat was tested 3 times at each pressure level, 20 s each dilation, with a 5-min interval; the average of the three tests was taken as the final result. The scoring criteria were as follows: 0 points, no behavioral response; 1 point, occidental head movements at the beginning of stimulation, while rats maintained still during the test; 2 points, slight contraction of abdominal muscles without rising of the abdomen; 3 points, strong contraction of abdominal muscles with notable rising of the abdomen but not the pelvis and scrotum; 4 points, the abdomen arched with rising of the pelvis and scrotum [66].

#### MWT test

The MWT test used von Frey filaments (Stoelting, IL, USA) to stimulate the center of the rat’s hind paw, not lasting over 4 s each time. Positive reactions were defined as lifting or licking the paw [67]. When the stimulation failed to induce a positive response, a one-grade higher stimulation would be applied; when the first positive reaction was induced, a one-grade lower stimulation would be applied. Each rat was tested 5 times at each grade of stimulation, with a 30 s interval. The bending force varied from 2.0 to 4.0, 6.0, 8.0, and 15.0 g, and the 50% MWT of the positive reaction was calculated using the up-down method.

#### TWL test

According to Hargreaves’s [68] method, the TWL test adopted a thermal stimulator (Institute of Medical Biology of Chinese Academy of Medical Sciences, Yunan, China) to observe the time from the beginning of thermal radiation till the appearance of paw lifting. Each rat was tested 3 times at a 3 min interval, and the average value was obtained as the final result.

### Sample processing

Rats were anesthetized using intraperitoneal injection of pentobarbital sodium and sacrificed via abdominal aortic blood collection. Rats were fixed in a supine position to separate the cecum, colon, and rectum. The part of the intestine from 2 cm above the anus to the end of the cecum was collected, cut along the mesenterium, and rinsed using normal saline at 4 ℃. One-centimeter notably injured part of the colon was collected and fixed in 4% paraformaldehyde. Then, rats were fixed in a prone position to expose the spinal cord and collect the L_6_-S_2_ segment by separating muscles on both sides of the spine, cutting cervical vertebral bodies, and slowly cutting vertebral pedicles perpendicularly from the broken end of the spinal canal. The collected spinal cord tissues were kept at -80 ℃.

### Histomorphological observation of colonic tissues

Histomorphological changes in colonic tissues were observed under a light microscope (Olympus, Toyko, Japan) using HE staining. The pathological injury score of colon was determined according to the scoring criteria (Supplementary Table 5) and the number of inflammatory cell was counted.

### High-throughput sequencing (RNA-seq)

Total RNA extraction: Each group contributed the entire segment of L_6_-S_2_ spinal cord tissues from 3 rats to extract the total RNA using the tissue RNA purification kit plus (EZBioscience, MN, USA). The purity and content were determined by an ultraviolet spectrophotometer NanoDrop 2000. The Agilent 4200 TapeStation system (Agilent, CA, USA) was used to test the quality of the total RNA, and the qualified samples would go through RNA-seq.

Establishing the full-transcriptome sequencing library: Removal of rRNA using the total RNA-seq library prep kit (Illumina, CA, USA); RNA fragmentation; first-strand cDNA synthesis during reverse transcription; replacing dTTP with dUTP during the second-strand cDNA synthesis while reserving the first strand; amplifying the first strand after purification, modification, and fragment length screening to construct the cDNA library for circRNA and mRNA.

Establishing the microRNA sequencing library: The gel electrophoresis was used to break 5 µL total RNA into 18–30 nt RNA fragments, amplified after end repair to establish the cDNA library for miRNA.

Sequencing and data analysis: Took 1 µL sample from each library to quality testing with the Agilent 2100 chip (Agilent, CA, USA). The qualified samples were then sent for sequencing using the Illumina sequencer (Illumina, CA, USA). We used HISAT2 to run sequence comparisons between CleanReads and the designated reference genome and collect the locations of the reference genome or genes, as well as the sequencing features of the sample [69]. The circRNA expression was calculated using the number of reads per million (RPM) clean tags, and the expression of miRNA was described using the transcript per million (TPM). Finally, the fragments per kilobase of exon model per million mapped fragments (FPKM) was used to normalize mRNA expression.

The raw sequence data reported in this paper have been deposited in the Genome Sequence Archive (Genomics, Proteomics & Bioinformatics 2021) in National Genomics Data Center (Nucleic Acids Res 2022), China National Center for Bioinformation / Beijing Institute of Genomics, Chinese Academy of Sciences (GSA: CRA015638) that are publicly accessible at https://ngdc.cncb.ac.cn/gsa [70, 71].

### Screening for DE genes and cluster analysis

The between-group difference was analyzed using DeSeq and EdgeR, and the expression difference of a gene between different samples was assessed using fold change (FC) and P-value. The expression difference was confirmed significant when *P*<0.05 and log_2_FC>1. Cluster 3.0 and Treeview were adopted to run cluster analysis for DE genes.

### Constructing circRNA-miRNA-mRNA ceRNA networks

We used databases such as miRanda to predict the regulation relationships between miRNA and circRNA, miRNA and mRNA, circRNA and mRNA, and establish circRNA-miRNA, miRNA-mRNA, and circRNA-mRNA pairs. Based on the Pearson correlation analysis, pairs with a correlation efficient *r* ≥ 0.80 and *P* ≤ 0.05 were selected; the default in miRanda (v3.3a) was used to predict the binding between sequences. We used ceRNA MuTATE to score ceRNA pairs and calculated the probability of ceRNA pairs sharing certain miRNAs with the hypergeometric distribution mathematic model. The Cytoscape 3.6.1 software was employed to visualize the corresponding regulation networks.

### GO and KEGG analysis of DE target mRNAs

The pathway enrichment analysis for DE mRNAs was run using the KEGG (Kyoto Encyclopedia of Genes and Genomes) database (http://www.genome.ad.jp/kegg); the function enrichment was analyzed using the GO (Gene Ontology) database (http://www.geneontology.org)[72, 73].

### Quantitative real-time PCR (qRT-PCR)

According to the Trizol kit (Invitrogen, CA, USA) instruction, the total RNA in rat spinal cord tissues was extracted and then detected for its content using the ND-1000 Nanodrop (Thermo Fisher, MA, USA). The cDNA of miRNA was generated using the miRNA reverse transcription kit (EZBioscience, MN, USA). The amplification reaction conditions were: 95 ℃ 5 min; 95 ℃ 10 s; 60 ℃ 30 s; 95 ℃ 15 s; 60 ℃ 1 min; 95 ℃ 30 s, 40 cycles. The cDNA of circRNA was synthesized using the PrimeScript™ RT Master Mix (Takara, Shiga, Japan) under: 95 ℃ 30 s, 1 cycle; 95 ℃ 5 s; 60 ℃ 30 s, 40 cycles; 95 ℃ 5 s; 60 ℃ 1 min, 1 cycle; 50 ℃ 30 s, 1 cycle. All amplification reactions were completed by the LightCycler 480 real-time quantitative PCR instrument (Roche, Basel, Switzerland). The primer sequences are shown in Supplementary Table 6. The relative amount was calculated using 2^ΔΔCt^; ΔCt = Target gene Ct value – reference gene Ct value. The 2^ΔΔCt^ of each sample = 2^ΔCt^ of each sample/the mean 2^ΔCt^ of all samples in the NG.

### Statistical analysis

SPSS 24.0 was used for statistical analyses. The data conforming to normal distribution were expressed as mean ± standard deviation (*x* ± *s*), and those not were described as median and quartiles [Median(P25, P75)]. The data conforming to normal distribution and homogeneity of variance were checked by the one-way ANOVA; the LSD for between-group comparisons and Bonferroni’s multiple comparison test for multiple comparisons. The data not in normal distribution or homogeneity of variance were processed using the Kruskal-Wallis H test, and the Games-Howell test for between-group comparisons. We took α = 0.05 as the significance level and confirmed statistical significance when *P*<0.05.

**Figures and legends**.

### Electronic supplementary material

Below is the link to the electronic supplementary material.

Supplementary Material 1

## Data Availability

The data that support the findings in the current study are available from the corresponding author upon reasonable request.
