# Supplementary material for: Moxibustion ameliorates chronic inflammatory visceral pain via spinal circRNA-miRNA-mRNA networks: a central mechanism study
Source: Mol Brain. 2024 May 15;17:23. doi: 10.1186/s13041-024-01093-7 (PMC11097453; doi:10.1186/s13041-024-01093-7)
Supplement: Supplementary file 1 — Supplementary Material 1 [file 13041_2024_1093_MOESM1_ESM.docx]

**Supplementary Tab. 1 CircRNA-miRNA-mRNA pairs**

| circRNA_id | Target miRNA/mRNA |
| --- | --- |
| circRNA_05361, circRNA_05547, circRNA_06739, circRNA_09943 | rno-miR-203a-3p /LOC100910207, Htr3a, Mcoln2, Aurkb, LOC108348139, Slc4a5 |
| circRNA_01290, circRNA_01614, circRNA_04991, circRNA_05361, circRNA_05547, circRNA_06103 | rno-miR-214-3p /Lrrc43, LOC108348139, Fgf21, Hoxd10 |
| circRNA_01290, circRNA_01614, circRNA_05361, circRNA_05547, circRNA_07183, circRNA_08139 | rno-miR-3120/Cpg1 |
| circRNA_00362, circRNA_01290, circRNA_02767, circRNA_04320, circRNA_04991, circRNA_05348, circRNA_05361, circRNA_05547, circRNA_07889, circRNA_08139, circRNA_11043, circRNA_11928 | rno-miR-483-3p /Lrrc43, Gfap, Slc2a3 |
| circRNA_01290, circRNA_05348, circRNA_05361, circRNA_05547 | rno-miR-483-5p/Hoxd10, LOC108348139 |

**Supplementary Tab. 2** **Prediction of circRNA-miRNA pairs**

| circRNA_id | Target miRNA | r | Total Score |
| --- | --- | --- | --- |
| circRNA_06103\|Chr2:116586880_116598683_+ | rno-miR-193a-3p | -0.82 | 443 |
| circRNA_00907\|Chr1:204960841_204980121_+ | rno-miR-193a-3p | -0.89 | 144 |
| circRNA_09943\|Chr6:86849959_86873166_+ | rno-miR-200b-3p | -0.86 | 597 |
| circRNA_09943\|Chr6:86849959_86873166_+ | rno-miR-200c-3p | -0.84 | 443 |
| circRNA_05361\|Chr18:85694201_85731039_- | rno-miR-203a-3p | -0.89 | 2031 |
| circRNA_09943\|Chr6:86849959_86873166_+ | rno-miR-203a-3p | -0.88 | 1513 |
| circRNA_06739\|Chr20:5377360_5416741_+ | rno-miR-203a-3p | -0.86 | 1337 |
| circRNA_05547\|Chr19:39164242_39195019_+ | rno-miR-203a-3p | -0.89 | 571 |
| circRNA_01290\|Chr1:278679022_278773472_+ | rno-miR-203a-3p | -0.83 | 420 |
| circRNA_11628\|Chr8:109243253_109288160_+ | rno-miR-203a-3p | -0.89 | 286 |
| circRNA_05361\|Chr18:85694201_85731039_- | rno-miR-214-3p | -0.86 | 874 |
| circRNA_04991\|Chr18:5191058_5285225_- | rno-miR-214-3p | -0.85 | 568 |
| circRNA_01290\|Chr1:278679022_278773472_+ | rno-miR-214-3p | -0.84 | 432 |
| circRNA_05547\|Chr19:39164242_39195019_+ | rno-miR-214-3p | -0.85 | 427 |
| circRNA_06103\|Chr2:116586880_116598683_+ | rno-miR-214-3p | -0.84 | 142 |
| circRNA_01614\|Chr10:57505175_57541484_+ | rno-miR-214-3p | -0.84 | 140 |
| circRNA_08139\|Chr4:65585304_65613754_+ | rno-miR-3120 | -0.88 | 1184 |
| circRNA_01290\|Chr1:278679022_278773472_+ | rno-miR-3120 | -0.86 | 448 |
| circRNA_07183\|Chr3:25875450_25942657_- | rno-miR-3120 | -0.83 | 305 |
| circRNA_05361\|Chr18:85694201_85731039_- | rno-miR-3120 | -0.89 | 288 |
| circRNA_05547\|Chr19:39164242_39195019_+ | rno-miR-3120 | -0.89 | 158 |
| circRNA_01614\|Chr10:57505175_57541484_+ | rno-miR-3120 | -0.89 | 141 |
| circRNA_01290\|Chr1:278679022_278773472_+ | rno-miR-451-5p | -0.83 | 281 |
| circRNA_04991\|Chr18:5191058_5285225_- | rno-miR-483-3p | -0.82 | 459 |
| circRNA_05547\|Chr19:39164242_39195019_+ | rno-miR-483-3p | -0.87 | 455 |
| circRNA_05361\|Chr18:85694201_85731039_- | rno-miR-483-3p | -0.86 | 425 |
| circRNA_00362\|Chr1:99532444_99539093_+ | rno-miR-483-3p | -0.90 | 289 |
| circRNA_05348\|Chr18:80660199_80668847_- | rno-miR-483-3p | -0.88 | 280 |
| circRNA_11928\|Chr9:28659251_28664255_- | rno-miR-483-3p | -0.87 | 164 |
| circRNA_02767\|Chr13:31140421_31152907_+ | rno-miR-483-3p | -0.82 | 151 |
| circRNA_11043\|Chr8:22600830_22606721_+ | rno-miR-483-3p | -0.90 | 144 |
| circRNA_08139\|Chr4:65585304_65613754_+ | rno-miR-483-3p | -0.89 | 142 |
| circRNA_01290\|Chr1:278679022_278773472_+ | rno-miR-483-3p | -0.82 | 141 |
| circRNA_04320\|Chr16:49367616_49369496_+ | rno-miR-483-3p | -0.83 | 140 |
| circRNA_07889\|Chr4:8162130_8166216_+ | rno-miR-483-3p | -0.88 | 140 |
| circRNA_05361\|Chr18:85694201_85731039_- | rno-miR-483-5p | -0.83 | 435 |
| circRNA_01290\|Chr1:278679022_278773472_+ | rno-miR-483-5p | -0.85 | 152 |
| circRNA_05348\|Chr18:80660199_80668847_- | rno-miR-483-5p | -0.83 | 142 |
| circRNA_05547\|Chr19:39164242_39195019_+ | rno-miR-483-5p | -0.82 | 142 |

**Supplementary Tab. 3** **Prediction of circRNA-miRNA pairs**

| circRNA_id | Target miRNA | r | Total Score |
| --- | --- | --- | --- |
| circRNA_06103\|Chr2:116586880_116598683_+ | rno-miR-193a-3p | -0.82 | 443 |
| circRNA_00907\|Chr1:204960841_204980121_+ | rno-miR-193a-3p | -0.89 | 144 |
| circRNA_09943\|Chr6:86849959_86873166_+ | rno-miR-200b-3p | -0.86 | 597 |
| circRNA_09943\|Chr6:86849959_86873166_+ | rno-miR-200c-3p | -0.84 | 443 |
| circRNA_05361\|Chr18:85694201_85731039_- | rno-miR-203a-3p | -0.89 | 2031 |
| circRNA_09943\|Chr6:86849959_86873166_+ | rno-miR-203a-3p | -0.88 | 1513 |
| circRNA_06739\|Chr20:5377360_5416741_+ | rno-miR-203a-3p | -0.86 | 1337 |
| circRNA_05547\|Chr19:39164242_39195019_+ | rno-miR-203a-3p | -0.89 | 571 |
| circRNA_01290\|Chr1:278679022_278773472_+ | rno-miR-203a-3p | -0.83 | 420 |
| circRNA_11628\|Chr8:109243253_109288160_+ | rno-miR-203a-3p | -0.89 | 286 |
| circRNA_05361\|Chr18:85694201_85731039_- | rno-miR-214-3p | -0.86 | 874 |
| circRNA_04991\|Chr18:5191058_5285225_- | rno-miR-214-3p | -0.85 | 568 |
| circRNA_01290\|Chr1:278679022_278773472_+ | rno-miR-214-3p | -0.84 | 432 |
| circRNA_05547\|Chr19:39164242_39195019_+ | rno-miR-214-3p | -0.85 | 427 |
| circRNA_06103\|Chr2:116586880_116598683_+ | rno-miR-214-3p | -0.84 | 142 |
| circRNA_01614\|Chr10:57505175_57541484_+ | rno-miR-214-3p | -0.84 | 140 |
| circRNA_08139\|Chr4:65585304_65613754_+ | rno-miR-3120 | -0.88 | 1184 |
| circRNA_01290\|Chr1:278679022_278773472_+ | rno-miR-3120 | -0.86 | 448 |
| circRNA_07183\|Chr3:25875450_25942657_- | rno-miR-3120 | -0.83 | 305 |
| circRNA_05361\|Chr18:85694201_85731039_- | rno-miR-3120 | -0.89 | 288 |
| circRNA_05547\|Chr19:39164242_39195019_+ | rno-miR-3120 | -0.89 | 158 |
| circRNA_01614\|Chr10:57505175_57541484_+ | rno-miR-3120 | -0.89 | 141 |
| circRNA_01290\|Chr1:278679022_278773472_+ | rno-miR-451-5p | -0.83 | 281 |
| circRNA_04991\|Chr18:5191058_5285225_- | rno-miR-483-3p | -0.82 | 459 |
| circRNA_05547\|Chr19:39164242_39195019_+ | rno-miR-483-3p | -0.87 | 455 |
| circRNA_05361\|Chr18:85694201_85731039_- | rno-miR-483-3p | -0.86 | 425 |
| circRNA_00362\|Chr1:99532444_99539093_+ | rno-miR-483-3p | -0.90 | 289 |
| circRNA_05348\|Chr18:80660199_80668847_- | rno-miR-483-3p | -0.88 | 280 |
| circRNA_11928\|Chr9:28659251_28664255_- | rno-miR-483-3p | -0.87 | 164 |
| circRNA_02767\|Chr13:31140421_31152907_+ | rno-miR-483-3p | -0.82 | 151 |
| circRNA_11043\|Chr8:22600830_22606721_+ | rno-miR-483-3p | -0.90 | 144 |
| circRNA_08139\|Chr4:65585304_65613754_+ | rno-miR-483-3p | -0.89 | 142 |
| circRNA_01290\|Chr1:278679022_278773472_+ | rno-miR-483-3p | -0.82 | 141 |
| circRNA_04320\|Chr16:49367616_49369496_+ | rno-miR-483-3p | -0.83 | 140 |
| circRNA_07889\|Chr4:8162130_8166216_+ | rno-miR-483-3p | -0.88 | 140 |
| circRNA_05361\|Chr18:85694201_85731039_- | rno-miR-483-5p | -0.83 | 435 |
| circRNA_01290\|Chr1:278679022_278773472_+ | rno-miR-483-5p | -0.85 | 152 |
| circRNA_05348\|Chr18:80660199_80668847_- | rno-miR-483-5p | -0.83 | 142 |
| circRNA_05547\|Chr19:39164242_39195019_+ | rno-miR-483-5p | -0.82 | 142 |

**Supplementary Tab. 4** **Prediction of miRNA-mRNA pairs**

| miRNA_id | Target mRNA | r | p-value | Total Score | MER |
| --- | --- | --- | --- | --- | --- |
| rno-miR-203a-3p | Pappa2 | -0.9021 | 0.0053 | 440 | 3 |
|  | Lin28b | -0.8327 | 0.0024 | 320 | 2 |
|  | Fat2 | -0.8726 | 0.0132 | 303 | 2 |
|  | Xkr7 | -0.9283 | 0.0495 | 297 | 2 |
|  | Polq | -0.9002 | 0.0035 | 157 | 1 |
|  | Sult2b1 | -0.8372 | 0.0377 | 156 | 1 |
|  | Aurkb | -0.9543 | 0.0076 | 155 | 1 |
|  | Nipal4 | -0.8472 | 0.0118 | 155 | 1 |
|  | Pmp2 | -0.9482 | 0.0070 | 148 | 1 |
|  | Zp2 | -0.9224 | 0.0404 | 147 | 1 |
|  | Depdc1b | -0.8904 | 0.0226 | 145 | 1 |
|  | Irs4 | -0.8937 | 0.0136 | 144 | 1 |
|  | Rtkn2 | -0.8467 | 0.0059 | 144 | 1 |
|  | Htr3a | -0.8224 | 0.0112 | 141 | 1 |
|  | Hoxd11 | -0.9373 | 0.0002 | 140 | 1 |
|  | Mcoln2 | -0.9234 | 0.0428 | 140 | 1 |
|  | Mylk | -0.8277 | 0.0003 | 140 | 1 |
|  | Pld5 | -0.9083 | 0.0053 | 140 | 1 |
|  | Trpc7 | -0.9409 | 0.0051 | 140 | 1 |
| rno-miR-483-3p | Mylk | -0.9388 | 0.0107 | 762 | 5 |
|  | Kcnmb2 | -0.9313 | 0.0121 | 718 | 5 |
|  | Lrrc43 | -0.9326 | 0.0067 | 601 | 4 |
|  | Xkr7 | -0.9206 | 0.0105 | 589 | 4 |
|  | Lin28b | -0.9183 | 0.0002 | 585 | 4 |
|  | Rtkn2 | -0.9135 | 0.0477 | 443 | 3 |
|  | Pappa2 | -0.9093 | 0.0000 | 309 | 2 |
|  | Slc17a7 | -0.9089 | 0.0004 | 309 | 2 |
|  | Sult2b1 | -0.9017 | 0.0052 | 298 | 2 |
|  | Cpg1 | -0.8990 | 0.0173 | 294 | 2 |
|  | Srd5a2 | -0.8976 | 0.0001 | 292 | 2 |
|  | Dok7 | -0.8773 | 0.0372 | 151 | 1 |
|  | Trpc7 | -0.8651 | 0.0053 | 145 | 1 |
|  | Htr3a | -0.8637 | 0.0171 | 143 | 1 |
|  | Cidea | -0.8486 | 0.0004 | 142 | 1 |
|  | Grifin | -0.8438 | 0.0388 | 142 | 1 |
|  | Depdc1b | -0.8356 | 0.0051 | 140 | 1 |
|  | Gfap | -0.8322 | 0.0058 | 140 | 1 |
|  | Slc2a3 | -0.8316 | 0.0105 | 140 | 1 |
| rno-miR-214-3p | Fgf21 | -0.8453 | 0.0340 | 326 | 2 |
|  | LOC108348139 | -0.8549 | 0.0300 | 298 | 2 |
|  | Lrrc43 | -0.8845 | 0.0192 | 282 | 2 |
|  | Hoxd10 | -0.9349 | 0.0062 | 143 | 1 |
| rno-miR-3120 | Cpg1 | -0.8539 | 0.0305 | 147 | 1 |
| rno-miR-451-5p | LOC108348139 | -0.9023 | 0.0138 | 140 | 1 |
| rno-miR-483-5p | Hoxd10 | -0.9834 | 0.0004 | 157 | 1 |
|  | LOC108348139 | -0.8754 | 0.0223 | 141 | 1 |

**Supplementary Tab. 5** **Histopathological Scoring criteria**

| Histopathological manifestation | | Score |
| --- | --- | --- |
| Ulcer | No ulcer | 0 |
|  | Ulcer area<3 mm | 1 |
|  | Ulcer area>3 mm | 2 |
| Inflammation | No inflammation | 0 |
|  | Mild inflammation | 1 |
|  | Moderate inflammation | 2 |
|  | Severe inflammation | 3 |
| Lesion depth | No lesion | 0 |
|  | Submucosa | 1 |
|  | Muscularis propria | 2 |
|  | Serosal layer | 3 |
| Fibrosis | No fibrosis | 0 |
|  | Mild fibrosis | 1 |
|  | Severe fibrosis | 2 |

**Supplementary Tab. 6 qRT-PCR primer sequence**

| RNA_id | Category | Primer sequence (5'-3') |
| --- | --- | --- |
| GAPDH | mRNA | Forward-GGAGTCCACTGGTGTCTTCA  Reverse-GGGAACTGAGCAATTGGTGG |
| U6 | snRNA | Forward-CTCGCTTCGGCAGCACA  Reverse-AACGCTTCACGAATTTGCGT |
| rno-miR-203a-3p | miRNA | Forward-CGGGCGTGAAATGTTTAGGA  Reverse-CAGCCACAAAAGAGCACAAT |
| rno-miR-483-3p | miRNA | Forward-CGGGCCACTCCTCCCCTCCC  Reverse-CAGCCACAAAAGAGCACAAT |
| circRNA_09943 | circRNA | Forward-ACAAAGTGCTATGACAGTTTGCTG  Reverse-CCACCAACGACAGCTCCTTTA |
| circRNA_02767 | circRNA | Forward-AATGGCGGATTGTCTGGAACA  Reverse-AATTGAACTTGCCCCTTCACCT |
| Gfap | mRNA | Forward-CTCAAGGTCGCAGGTCAA  Reverse-GAGTGGTATCGGTCCAAGTT |
| Aurkb | mRNA | Forward-ACAACCTTCTTGCAGCTCCTC |
|  |  | Reverse-CCTTCTGACCCATACCCACC |
